# Bacterial Community Associated with the Reef Coral *Mussismilia braziliensis's* Momentum Boundary Layer over a Diel Cycle

**DOI:** 10.3389/fmicb.2017.00784

**Published:** 2017-05-22

**Authors:** Cynthia B. Silveira, Gustavo B. Gregoracci, Felipe H. Coutinho, Genivaldo G. Z. Silva, John M. Haggerty, Louisi S. de Oliveira, Anderson S. Cabral, Carlos E. Rezende, Cristiane C. Thompson, Ronaldo B. Francini-Filho, Robert A. Edwards, Elizabeth A. Dinsdale, Fabiano L. Thompson

**Affiliations:** ^1^Instituto de Biologia, Universidade Federal do Rio de JaneiroRio de Janeiro, Brazil; ^2^Department of Biology, San Diego State UniversitySan Diego, CA, USA; ^3^Universidade Federal de São Paulo - Baixada SantistaSantos, Brazil; ^4^Centre for Molecular and Biomolecular Informatics, Radboud Institute for Molecular Life Sciences, Radboud University Medical CentreNijmegen, Netherlands; ^5^Department of Computational Science, San Diego State UniversitySan Diego, CA, USA; ^6^Laboratório de Ciências Ambientais, Universidade Estadual do Norte FluminenseCampos dos Goytacazes, Brazil; ^7^Departamento de Engenharia e Meio Ambiente, Universidade Federal da ParaíbaRio Tinto, Brazil; ^8^Laboratório de Sistemas Avançados de Gestão da Produção, COPPE, Universidade Federal do Rio de JaneiroRio de Janeiro, Brazil

**Keywords:** coral reef microbiome, diurnal cycle, Abrolhos, coral momentum boundary layer, coral physiology, coral mucus

## Abstract

Corals display circadian physiological cycles, changing from autotrophy during the day to heterotrophy during the night. Such physiological transition offers distinct environments to the microbial community associated with corals: an oxygen-rich environment during daylight hours and an oxygen-depleted environment during the night. Most studies of coral reef microbes have been performed on samples taken during the day, representing a bias in the understanding of the composition and function of these communities. We hypothesized that coral circadian physiology alters the composition and function of microbial communities in reef boundary layers. Here, we analyzed microbial communities associated with the momentum boundary layer (MBL) of the Brazilian endemic reef coral *Mussismilia braziliensis* during a diurnal cycle, and compared them to the water column. We determined microbial abundance and nutrient concentration in samples taken within a few centimeters of the coral's surface every 6 h for 48 h, and sequenced microbial metagenomes from a subset of the samples. We found that dominant taxa and functions in the coral MBL community were stable over the time scale of our sampling, with no significant shifts between night and day samples. Interestingly, the two water column metagenomes sampled 1 m above the corals were also very similar to the MBL metagenomes. When all samples were analyzed together, nutrient concentration significantly explained 40% of the taxonomic dissimilarity among dominant genera in the community. Functional profiles were highly homogenous and not significantly predicted by any environmental variables measured. Our data indicated that water flow may overrule the effects of coral physiology in the MBL bacterial community, at the scale of centimeters, and suggested that sampling resolution at the scale of millimeters may be necessary to address diurnal variation in community composition.

## Introduction

The rules governing associations between bacteria and corals represent a current theme of debate (Thompson et al., 2015; Douglas and Werren, 2016; Theis et al., 2016). Temporal dynamics of bacterial community composition and functional roles in coral holobionts add important information to this debate (Garren and Azam, 2012; Bourne et al., 2016). The majority of studies on the changes in coral bacterial community composition over time have been focused on stress and disease conditions (Ritchie, 2006; Garren et al., 2009; Sweet et al., 2014; Zaneveld et al., 2016). However, coral-associated bacteria can change over coral animal developmental stages and natural mucus aging (Apprill et al., 2009; Sweet et al., 2011b; Sharp et al., 2012; Glasl et al., 2016). When colony size is utilized as a proxy for age, bacterial community diversity in the coral *Coelastrea aspera* increases in a step-wise pattern from juveniles to medium colonies, decreasing again in older, larger colonies (Williams et al., 2015). Short time scale changes in response to tides have also been reported in *C. aspera*, and are dependent of colony age (Sweet et al., 2017). Understanding the dynamics of coral-microbe associations over time is necessary in order to predict coral's response to disturbance and design manipulation strategies to improve coral resilience (Ainsworth and Gates, 2016; Sweet and Brown, 2016).

Corals experience fundamental physiological changes in response to light availability over day–night cycles. They switch from autotrophic holobionts during the day, when intracellular zooxanthellae produce carbon surpluses through photosynthesis, to heterotrophs during the night, when polyps prey on zooplankton (Shashar et al., 1993; Anthony and Fabricius, 2000). Animal tissue respiration is higher at night and early morning compared to the afternoon due to digestion of prey captured at night (Schneider et al., 2009). This physiologic switch results in hyperoxic conditions in the holobiont during the day, and hypoxia during the night, with the polyp oral cavity becoming almost anoxic (Shashar et al., 1993; Haas et al., 2013). Coral respiration and calcification also reduces the pH of the surrounding environment during the night (Smith et al., 2013). Coral's molecular responses to light, pH, and redox potential changes over the day includes overexpression of cryptochromes, antioxidant enzymes such as catalase and carbonic anhydrase, which may explain light-enhanced coral calcification (Chalker and Taylor, 1975; Levy et al., 2011). Increased expression of protective enzymes against reactive oxygen species, such as superoxide dismutase and catalase is reflected in enhanced activity of these enzymes during the day, a protective mechanism for the cnidarian-zooxanthellae association (Levy et al., 2006). Enzymes involved in glycolysis and several biosynthetic pathways are among the ones overexpressed during the night (Levy et al., 2011). Diel transcriptomic patterns of photoreceptors, putative circadian regulation genes, stress response genes, and metabolic genes were also observed in the coral *Acropora cervicornis* (Hemond and Vollmer, 2015). The production of microsporine-like aminoacids follows sunlight cycles, and it is speculated to protect corals from ultraviolet radiation damage (Yakovleva and Hidaka, 2004). Microbes recycle up to 45% of the carbon fixed by *Symbiodinium* and exuded as coral mucus, highlighting bacterial dependence on coral physiology for growth (Brown and Bythell, 2005). Changes in oxygen availability and pH in coral tissues are expected to reflect changes in microbial community composition and metabolism. Nevertheless, virtually all studies on coral-associated and reef microbiomes are based on samples taken during the day due to logistical restrictions. This sampling bias could heavily distort the understanding of microbial community composition and function in the coral holobiont.

Biological rhythms often account for periodicity in host–parasite interactions in many animal models (Martinez-Bakker and Helm, 2015). In corals, diel physiological changes are important in black band disease development by interfering with the metabolism of coral-associated Cyanobacteria (Carlton and Richardson, 1995). Although anoxic conditions are consistently maintained at the coral–mat interface, sulfide production at night accelerates band progression by creating a toxic environment for coral tissues (Carlton and Richardson, 1995). In an analogous case, compounds produced during the day by symbiotic zooxanthellae, such as dimethylsulfoniopropionate (DMSP) and dimethylsulfide (DMS), intermediate coral-microbe associations (Raina et al., 2010). In the coral *Pocillopora damicornis*, DMSP functions as a chemotaxis and chemokinesis cue for the potential pathogen *Vibrio corallilyticus* (Garren et al., 2014). Diurnal changes in pH are also predicted to alter the coral-associated microbiota. Sustained reduced pH, simulating ocean acidification scenarios, lead to distinct bacterial community composition with implications for coral physiological adaptation (Meron et al., 2011; Webster et al., 2013; O'Brien et al., 2016). These studies indicate that under stress conditions, normal diurnal physiological cycles may interfere with the progression of disease and coral survival, particularly in cases of polymicrobial diseases (Bourne et al., 2009; Sweet and Bulling, 2017).

Corals influence the reef overlying water and surrounding sediments through the release of organic carbon-rich mucus (Wild et al., 2004, 2005; Bythell and Wild, 2011). The level of influence corals have on the water is dependent of the mucus composition and solubility, ranging from particulate material that serve as particle traps to dissolved labile molecules readily utilized for bacterial growth (Wild et al., 2004; Haas et al., 2011). Mucus constantly released by corals and benthic physiologic processes create three boundary layers over the reef benthos: the first, called diffusive boundary layer, is millimeters thick and is formed by the diffusion of benthic metabolism, such as photosynthesis and respiration; the second, called momentum boundary layer (MBL), is a viscous sublayer centimeters thick, and is formed by a combination of products released by the benthos and the friction of water movement over corals; the third, called benthic boundary layer, is about 1 m thick, is formed by water turbulence and controls interactions of the reef with the open sea waters (Shashar et al., 1993, 1996; Barott and Rohwer, 2012). Coral mucus release rate and mucus composition varies in response to light. In *Acropora acuminata*, the lipid content varies between night and day in response to photosynthesis rates by zooxanthellae, as the endosymbiotic algae are the primary sites of mucus lipid production (Crossland, 1987). Therefore, coral influence on benthic boundary layers is predicted to differ between day and night. Water column reef communities experience diurnal physiological changes in response to photosynthesis/respiration rates (Kayanne et al., 1995). Yet, these changes are thought to be a result of benthic respiration and calcification, as most reef production is of benthic origin (Kayanne et al., 1995; Silveira et al., 2015). The water column microbial communities in Heron Island, Great Barrier Reef, was significantly different between day and night (Sweet et al., 2010). However, the lack of differences between tides was interpreted as a weak connection between the benthos and the water column, with planktonic processes governing the differences observed or, alternatively, rapid turnover and mixing offsetting the benthic–pelagic coupling (Sweet et al., 2010).

Here, we hypothesized that the microbial community within coral MBL responds to changes in coral physiology. We investigated the microbial community associated with the MBL over the Brazilian endemic reef coral *Mussismilia braziliensis* in response to light availability. This genus forms ~70% of the reef structure in the Abrolhos and is highly adapted to high turbidity stress periodically observed in the region due to continental runoff and sediment resuspension (Leão and Kikuchi, 2005; Segal et al., 2012; Loiola et al., 2013). *Proteobacteria, Bacteroidetes, Firmicutes, Cyanobacteria*, and *Actinomycetes* are the main groups associated to *M. braziliensis* (Reis et al., 2009; Castro et al., 2010; Garcia et al., 2013). In the present study, microbial community composition and functional profiles were compared between *M. braziliensis*'s MBL and water column over one diel cycle. Microbial metagenomes were also compared with changes in nutrient concentrations and microbial, viral and picoeukaryotic abundances. We found that the coral MBL did not display a diurnal pattern in the time scale analyzed. The similarity between MBL and water column, and the correlation with nutrient concentrations suggested that water flow over corals outweighs the effect of coral physiology on the MBL microbial community.

## Materials and methods

### Sampling

Samples were collected from Santa Bárbara island (17.9647778S, 38.7027778W), inside the Abrolhos Marine National Park (SISBIO permit 27147-2) in August 14th to 16th, 2011, during the austral winter. The main organisms contributing to benthic cover in Santa Bárbara are: coral 7.7%, crustose coralline algae (CCA) 6.6%, fleshy algae 22.4%, and turf algae 66.4%. Our sampling strategy targeted the MBL above visually healthy *M. braziliensis* corals. MBL samples were taken within 5 cm from the coral surface by scuba divers using a manual bilge pump (Haas et al., 2014). To minimize the amount of overlying water into the MBL sample, material was pumped from the surface of eight adjacent colonies at each time point, for a total of 80 l. Due to the sampling method, this sample was mainly composed of coral MBL over the corals, but include some benthic boundary layer (further above the corals) and coral mucus that detaches from the colony (Barott and Rohwer, 2012). Water column samples were taken from 1 m above the corals. Coral's MBL and water column were sampled at 5 and 4 m depth, respectively. Corals were sampled every 6 h for 48 h starting at 12:00 h on August 14th, for a total of nine samples through time. Water column was sampled every 6 h for 24 h starting at the same time, for a total of five samples through time. The reef area where the sampled coral heads were located was marked so we could return to the same location each time. Samples for nutrient and metagenomic analysis were collected and preserved according to Haas et al. (2014). From each sample, three 50 ml aliquots were flash frozen in liquid nitrogen for nutrient concentration analysis. For Chlorophyll *a* analysis, triplicate 2 l subsamples were immediately filtered through 0.45 μm pore-size ester-cellulose filters (Millipore) and the filters were frozen. Three 2 l subsamples were filtered through 0.7 μm pore GF/F glass fiber filter and aliquots of 50 ml of the filtrate were conserved in amber bottles with 1 ml of phosphoric acid for Dissolved Organic Carbon (DOC) determination (Rezende et al., 2010). Triplicate 1 ml subsamples were fixed with paraformaldehyde, glutaraldehyde and a mix of paraformaldehyde and glutaraldehyde (1, 0.5, and 1 + 0.05% final concentrations) for the fixation of eukaryotic autotrophs, viruses and bacteria, respectively. Sixty liters of each sample were concentrated through 100 kDa tangential flow filtration and subsequently filtered through a 0.22 μm Sterivex to concentrate microbes. Sterivex were frozen in liquid nitrogen until laboratory processing for metagenomics sequencing.

### Nutrients and microbial abundance measurements

Inorganic nutrients concentrations were determined using standard oceanographic methods (Grasshoff et al., 2009). Briefly: (1) ammonia was determined by indophenol, (2) nitrite was determined by diazotization, (3) nitrate was determined by reduction in Cd–Cu column followed by diazotization, (4) total nitrogen was determined by digestion with potassium persulfate following nitrate determination, (5) orthophosphate was determined by reaction with ascorbic acid, (6) total phosphorous by acid digestion to phosphate, and (7) silicate by reaction with molybdate. DOC concentration in the filtrates was determined by high catalytic oxidation using a TOC/TDN analyser (Shimadzu) (Rezende et al., 2010). Chlorophyll filters were extracted overnight in 90% acetone at 4°C and analyzed by fluorometry for chlorophyll concentration using a Turner Designs TD-700 fluorometer (Sunnyvale, CA, USA). Prokaryotic and viral abundances were determined by flow cytometry after incubating the samples with SYBR Green nucleic acid staining (Life Technologies, Carlsbad, CA) as previously described (Andrade et al., 2003). Autotrophic eukaryotes abundance (pico and nanoeukaryotes) was determined by flow cytometry through chlorophyll autofluorescence.

### Metagenomic sequencing and anotation

To investigate the composition of microbial assemblages associated to *M. braziliensis* MBL we sequenced three metagenomes at “dark” time points: 00:00 on August 15th, 00:00 on August 16th, and 06:00 on August 16th (at the end of the second night). Three “day” time points were sequenced: 18:00 on August 14th (the first sampling day), 18:00 on August 15th, and 12:00 on August 16th, the last sampling day. We also sequenced one “night” water column sample from 00:00 on August 15th and one “day” water column sample from 12:00 on August 15th. Samples from 18:00 were taken before sunset, at the end of 12 h of daylight exposure, and therefore were grouped as “day.” The same criteria was applied to 06:00 samples grouped as “night.”

DNA was extracted from Sterivex after proteinase K/SDS treatment by phenol/chloroform/isoamyl alcohol technique, and sequenced on an Ion Torrent sequencer (Life Sciences, USA). Reads shorter than 100 bp and with mean quality scores lower than 25 were removed using PrinSeq (Schmieder and Edwards, 2011b). High quality sequences were then de-replicated with TagCleaner (Schmieder et al., 2010) and potential contaminants matching lambda or human DNA sequences removed with DeconSeq (Schmieder and Edwards, 2011a). A summary of metagenomic sequencing, quality control, and sample grouping used in multivariate analyses is presented in Table 1. Taxonomic annotation was performed using FOCUS and functional annotation using SUPERFOCUS (Silva et al., 2014, 2016). Metagenomes are publically available on FigShare (https://doi.org/10.6084/m9.figshare.4614745.v1) and MG-RAST under the project *Diel Mussismilia* (http://metagenomics.anl.gov/linkin.cgi?project=mgp14437; Meyer et al., 2008). Taxonomic and functional annotation tables utilized in multivariate analysis are provided as online Supplementary Materials.

**Table 1:** **Metagenomes summary**.

**Sample**	**Time group**	**Sequences generated**	**Sequences after QC**	**Passed QC (%)**	**MG-RAST ID**
Coral MBL_08.15.11_18:00	Day	799,095	601,398	75.26	4644203.3
Coral MBL_08.15.11_00:00	Night	4,820,694	4,096,018	84.97	4644204.3
Coral MBL_08.15.11_18:00	Day	2,007,447	1,466,499	73.05	4644206.3
Coral MBL_08.16.11_00:00	Night	1,946,874	1,495,057	76.79	4644206.3
Coral MBL_08.16.11_06:00	Night	47,502	37,850	79.68	4644208.3
Coral MBL_08.16.11_12:00	Day	71,627	42,363	59.14	4644207.3
Water column_08.15.11_00:00	Night	1,397,498	1,114,569	79.75	4644209.3
Water column_08.15.11_12:00	Day	5,74,364	4,47,371	77.89	4644210.3

*Number of sequences generated and percentage of sequences that passed quality control using Prinseq. Sample source and time group indicate sample clustering for PERMANOVA-tests*.

### Statistical analysis

Statistical analyses were performed using Vegan package in R (Dixon, 2003) or using Primer 6 (PRIMER-E, Plymouth). Relative abundances (%) of taxonomic and functional groups were analyzed through non-metric multidimentional scaling (NMDS) in Vegan (Dixon, 2003). The presence of significant clusters (time or sample source) was determined using Permutational ANOVA (PERMANOVA) using Bray-Curtis dissimilarities (Anderson, 2001). To test for correlation between environmental variables and taxonomic and functional profiles we reduced the dataset to taxa or functions representing >1% of the annotated sequences (mean across all eight samples). The use of abundant groups is expected to reduce stochastic effects associated with rare members or functions (Shade and Gilbert, 2015). Distance-based linear models (DistLM) analysis using Bray Curtis dissimilarity and scaled environmental variables was used to determine the contribution of each environmental variable to the total dissimilarity on taxonomic and functional profiles (McArdle and Anderson, 2001). DistLM was ran stepwise with a second order Akaike Information Criteria (AICc) in Primer. We selected variables that contributed to 10% or more to the dissimilarity and performed a Canonical Correspondence Analysis (CCA) using Vegan in R to visualize the relationship between environmental variables and taxa/function. We performed a Pearson correlation test followed by Holm's *p*-values post-correction to determine significant correlations between environmental variables and taxa/functions (Holm, 1979). Microbial communities can be modulated by the available nutrients, but the opposite scenario, where nutrient availability is a product of microbial metabolism, is also possible. Therefore, we also tested taxonomic and functional profiles as predictor of environmental variables in a separate DistLM-test.

## Results

### Chlorophyll, organic, and inorganic nutrients

Variation in organic and inorganic forms of nitrogen and phosphorous over time are shown in Table 2. Concentration of total phosphorus and nitrogen, ammonium, and nitrite did not co-vary over time (Pearson correlation, *p* > 0.05), while orthophosphate and nitrate were positively correlated (Pearson correlation *r* = 0.72, *p* = 0.003). Nitrate contributed to most of the inorganic nitrogen pool both in the coral MBL and the water column (1.70 ± 0.04 μM, mean ± *SE* for coral and 1.79 ± 0.26 for water column, compared to 0.15 ± 0.05 and 0.05 ± 0.05 for nitrite and 0.17 ± 0.07 and 0.29 ± 0.14 for ammonium in coral and water column, respectively). Concentration of DOC and Chlorophyll *a* are shown in Table 2. DOC correlated with total nitrogen, including the organic nitrogen pool (Pearson correlation *r* = 0.79, *p* < 0.001). Chlorophyll *a*, DOC, and nutrient concentrations were not significantly different between day and night samples (Welch Two Sample *t*-test *p* > 0.05 for all tests on the coral MBL only, water column only, and MBL and water combined). Therefore, we were not able to identify diurnal patterns in nutrient concentration in the MBL above *M. braziliensis* or the water column at this time scale.

**Table 2:** **Inorganic nutrients, Chlorophyll a and DOC concentrations in ***M. braziliensis*** boundary layer and water column over a diel cycle**.

**Sample**	**Date**	**Time**	**Ortophosphate**	**Total Phosphorus**	**NH_3_**	**Nitrite**	**Nitrate**	**Total Nitrogen**	**Chlorophyll *a***	**DOC**
Coral MBL	08/14	12:00	0.12 (0.00)	0.29 (0.01)	0.05 (0.01)	0.05 (0.00)	1.69 (0.02)	5.61 (0.25)	0.06 (0.02)	115.8 (00.0)
Coral MBL	08/14	18:00	0.18 (0.01)	0.39 (0.01)	0.15 (0.03)	0.06 (0.01)	1.80 (0.01)	7.56 (0.83)	0.26 (0.03)	113.3 (15.8)
Coral MBL	08/15	00:00	0.10 (0.01)	0.22 (0.01)	0.42 (0.04)	0.05 (0.00)	1.63 (0.03)	6.77 (0.39)	0.46 (0.06)	129.1 (25.0)
Coral MBL	08/15	06:00	0.18 (0.01)	0.36 (0.01)	0.61 (0.09)	0.07 (0.00)	1.58 (0.02)	9.00 (1.75)	0.26 (0.06)	140.8 (37.5)
Coral MBL	08/15	12:00	0.14 (0.01)	0.37 (0.01)	0.05 (0.00)	0.08 (0.01)	1.75 (0.08)	11.24 (1.74)	1.62 (0.39)	155.8 (1.66)
Coral MBL	08/15	18:00	0.14 (0.01)	0.27 (0.01)	0.05 (0.00)	0.07 (0.01)	1.49 (0.11)	7.38 (0.48)	0.35 (0.03)	125.0 (39.1)
Coral MBL	08/16	00:00	0.73 (0.03)	na	na	0.47 (0.01)	1.69 (0.05)	7.7 (0.86)	0.62 (0.11)	129.1 (25.0)
Coral MBL	08/16	06:00	0.14 (0.00)	0.25 (0.01)	0.06 (0.01)	0.08 (0.02)	1.88 (0.07)	6.65 (0.57)	0.35 (0.17)	104.1 (3.33)
Coral MBL	08/16	12:00	4.07 (0.05)	na	0.08 (0.01)	0.41 (0.01)	na	7.7 (0.86)	0.45 (0.21)	105.8 (9.16)
Water Column	08/14	12:00	0.08 (0.00)	0.24 (0.01)	<0.05 (0.00)	0.04 (0.01)	1.72 (0.09)	5.09 (0.54)	0.31 (0.02)	169.1 (49.1)
Water Column	08/14	18:00	0.08 (0.00)	0.21 (0.01)	0.08 (0.01)	0.05 (0.01)	1.67 (0.02)	5.50 (0.69)	0.31 (0.24)	135.8 (45.0)
Water Column	08/15	00:00	0.11 (0.01)	0.23 (0.00)	1.00 (0.09)	0.05 (0.01)	1.82 (0.04)	5.17 (0.50)	0.30 (0.22)	103.3 (7.5)
Water Column	08/15	06:00	0.11 (0.02)	0.21 (0.01)	0.33 (0.04)	0.05 (0.00)	1.93 (0.08)	5.15 (0.01)	0.15 (0.04)	136.6 (67.5)
Water Column	08/15	12:00	0.09 (0.01)	0.21 (0.03)	0.05 (0.00)	0.06 (0.01)	1.81 (0.01)	5.08 (0.40)	0.30 (0.08)	114.1 (12.5)

*Values represent means of triplicate measurements, in μmol.l^-1^, and standard deviation in parenthesis*.

### Microbial abundance

**Figure 1:**
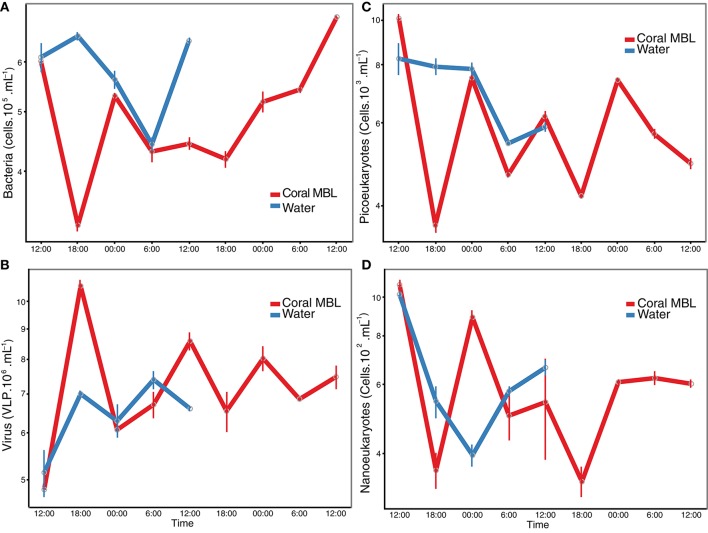
**Microbial abundance over time**. Abundance of **(A)** prokaryotic cells, **(B)** viral-like particles, autotrophic **(C)** pico- and **(D)** nanoeukaryotes was determined by flow cytometry. Red line indicates the coral momentum boundary layer and blue indicates the water column one meter above the corals.

Prokaryotic abundances ranged from 3.2 × 10^5^ to 7.0 × 10^5^ ml^−1^ in the coral MBL, and from 4.4 × 10^5^ to 6.6 × 10^5^ ml^−1^ in the water column. Viral abundances ranged from 4.8 × 10^6^ to 10.6 × 10^6^ ml^−1^ on the coral and 5.1 × 10^6^ to 7.3 × 10^6^ ml^−1^ in the water column. When analyzing MBL and water column samples together, viruses and prokaryotes showed a significant negative correlation to each other (Pearson *r* = −0.57, *p* = 0.03). Prokaryotic abundance also correlated negatively to total phosphorous concentration (Pearson *r* = −0.72, *p* = 0.003), while viral abundance correlated positively to total phosphorous (Pearson *r* = 0.56, *p* = 0.03). The abundance of autotrophic pico- and nanoeukayotes ranged from 4.2 × 10^3^ to 10 × 10^3^ ml^−1^ and from 3.6 × 10^2^ to 10.8 × 10^2^ ml^−1^, respectively. Pico and nanoeukaryote abundances were positively correlated to each other (Pearson *r* = 0.66, *p* = 0.008) and were both negatively correlated to viral abundance (Pearson *r* = −0.61, *p* = 0.01 and *r* = −0.65, *p* = 0.01 for pico- and nanoeukaryotes, respectively). Prokaryotic and picoeukaryote abundances correlated positively (Pearson *r* = 0.76, *p* = 0.001). No diurnal patterns were observed in the microbial and viral abundances on the coral MBL or in the water column (Welch Two Sample *t*-test *p* > 0.05 for all tests on the coral MBL only, water column only, and MBL and water combined). However, over the course of the diurnal monitoring we detected a viral burst event that occurred at 18:00 of the first night. At this time, prokaryotic abundances in the MBL dropped to the lowest values recorded during the cycles, while viral abundances peaked (Figure 1).

### Microbial taxonomic and functional profiles

The microbial community associated with *M. braziliensis* MBL at night was dominated by *Proteobacteria* (mean of 32.2% of annotated reads), *Firmicutes* (21.5%), *Thaumarchaeota* (9.6%), *Euryarchaeota* (8.4%), *Cyanobacteria* (5.9%), and *Bacteroidetes* (4.4%). During the day, the same phyla were dominant, except for *Euryarchaeota*: *Proteobacteria* (41.6%), *Bacteroidetes* (13.79%), *Cyanobacteria* (8.3%), *Firmicutes* (8.2%), *Thaumarchaeota* (4.3%), and *Tenericutes* (4.2%). The main phyla in the water column at night were *Proteobacteria* (39.4%), Cyanobacteria (17.2%), *Firmicutes* (9.4%), *Thaumarchaeota* (7.4%), and *Euryarchaota* (5.7%). Overall, the same phyla dominated all three groups of samples.

**Figure 2:**
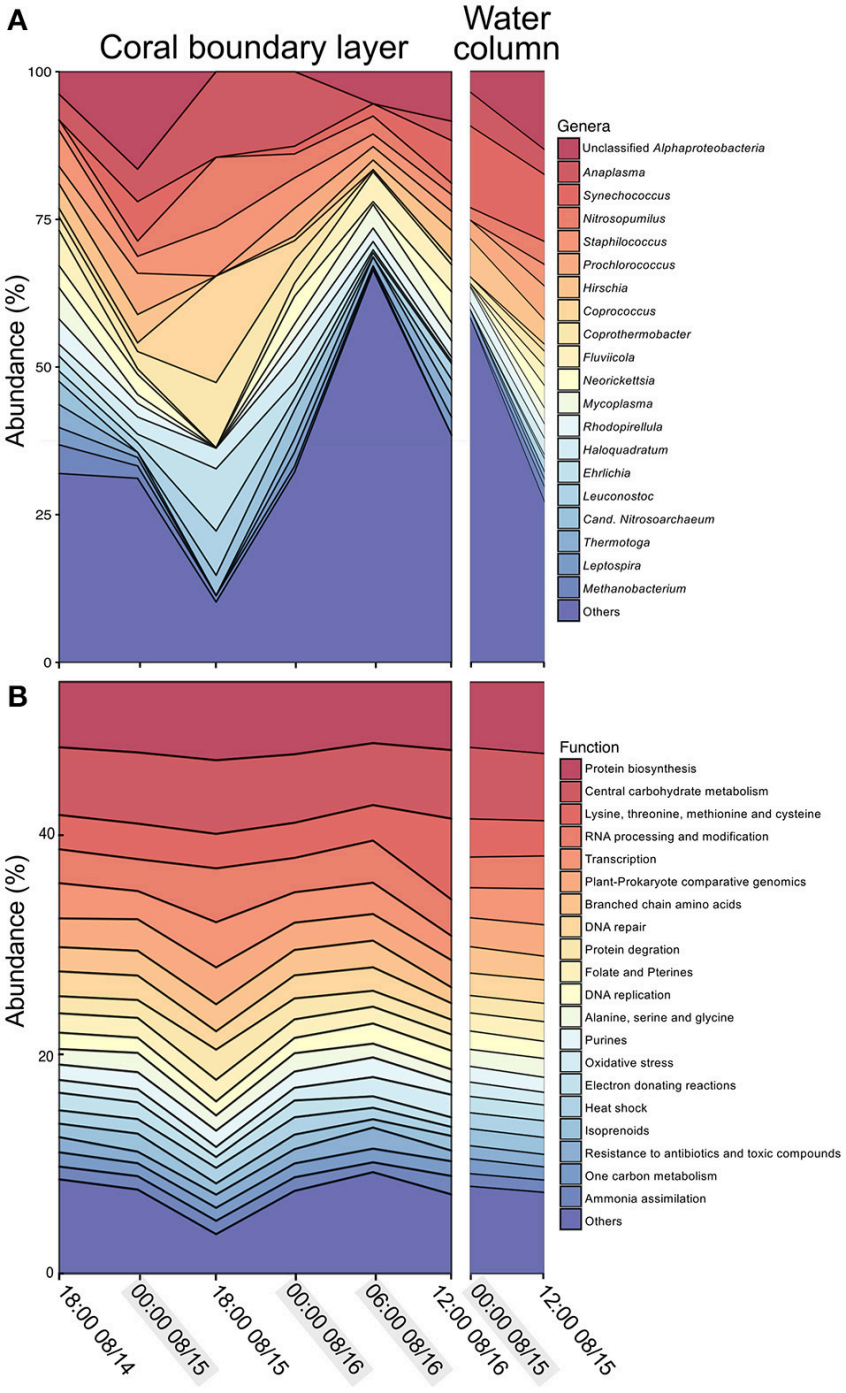
**Taxonomic and functional profiles**. Abundance of **(A)** dominant genera as assigned by FOCUS in the coral boundary layer (left panel) and water column (right panel), and **(B)** dominant level 2 Subsystem functions in SEED database as assigned by SUPERFOCUS the coral boundary layer (left panel) and water column (right panel). Shadowed time points indicate samples grouped as “night.”

**Figure 3:**
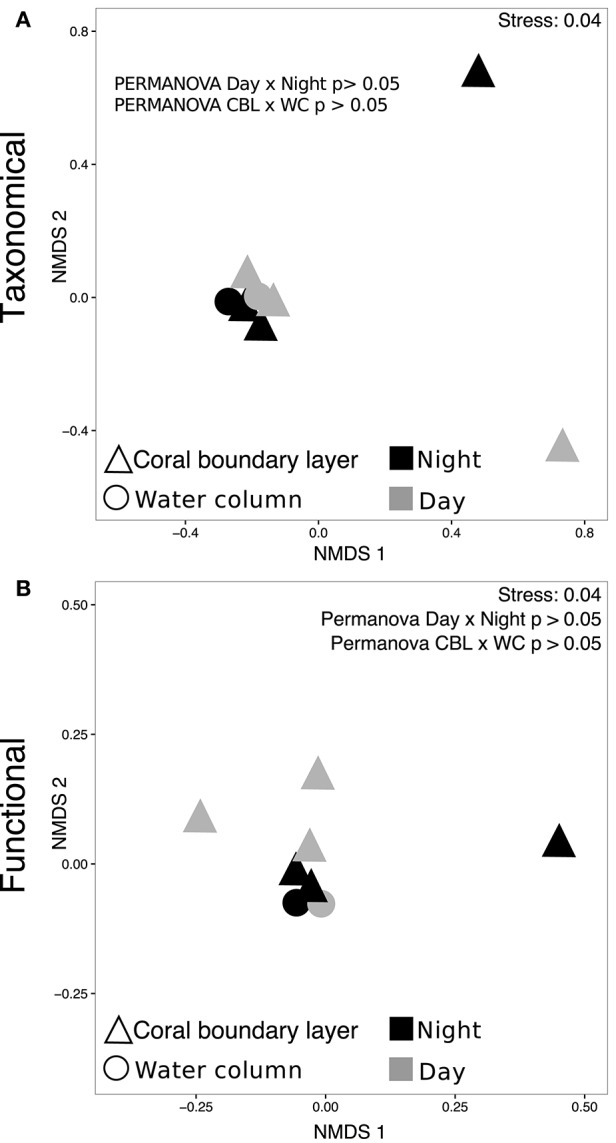
**Taxonomic and functional clustering**. NMDS plots and PERMANOVA tests based on the abundance of **(A)** genera and **(B)** level 2 Subsystems functions SEED database.

At the genus level, the microbial community associated with *M. braziliensis* MBL at night was dominated by members of the genera *Anaplasma* (9.2%, mean of annotated sequences), *Coprococcus* (6.05%), *Staphylococcus* (5.6%), unclassified *Alphaproteobacteria* (5.0%), *Coprothermobacter* (5.0%), and the archaeal genus *Nitrosopumilus* (5.0%) (Figure 2A and Supplementary Material 1). During the day, the dominant members were unclassified *Alphaproteobacteria* (6.9%), *Fluviicola* (4.8%), *Vibrio* (4.6%), *Synechococcus* (4.6%), *Glaciecola* (3.8%), and *Alteromonas* (3.5%). The most abundant genera in the water column were *Synechococcus* (12.5%), unclassified *Alphaproteobacteria* (8.3%), *Hirschia* (5.3%), *Anaplasma* (4.9%), *Prochorococcus* (4.4%), and *Nitrosopumilus* (3.0%). NMDS analysis based on relative abundances of genera showed no significant clusters among the three tested groups (water column, MBL daytime and MBL nighttime, PERMANOVA *p* > 0.05, Figure 3A). Bacterial richness, diversity, and evenness at the species level did not significantly differ between day and night in the MBL (Table 3, *t*-test *p* = 0.29, 0.347, and 0.0516 for Chao richness, Shannon diversity (*H*′), and Pielou's evenness, respectively).

**Table 3:** **Bacterial richness and diversity indexes at the species level**.

**Sample**	**Species count**	**Chao**	**Shannon (*H′*)**	**Evenness**
Coral MBL_08.15.11_18:00	1,363	1,402	5.527721	0.7658835
Coral MBL_08.15.11_00:00	1,446	1,456	4.906071	0.6742298
Coral MBL_08.15.11_18:00	1,088	1,245	5.510376	0.7880864
Coral MBL_08.16.11_00:00	1,403	1,424	5.238537	0.722919
Coral MBL_08.16.11_06:00	1,049	1,254	5.203073	0.7480417
Coral MBL_08.16.11_12:00	604	935	4.977831	0.7773519
Water column_08.15.11_00:00	1,408	1,424	5.145579	0.7097424
Water column_08.15.11_12:00	1,391	1,435	4.963443	0.6857689

Abundant functional subsystems were remarkably similar across MBL and water samples at night and day (Figure 2B and Supplementary Material 2). The most abundant functions at the level 1 Subsystems according to SEED database were carbohydrates (13.1 ± 0.5%, mean ± *SD*); amino acids and derivatives (11.9 ± 1.4%); protein metabolism (9.6 ± 1.2%); cofactors, vitamins, prosthetic groups, pigments (8.7 ± 0.5%), and RNA metabolism (6.3 ± 1.1%). At the level 2, most of the predicted proteins were unclassified (20.9 ± 1.5%, mean ± *SD*), followed by protein biosynthesis (6.3 ± 0.5%), central carbohydrate metabolism (6.3 ± 0.3%), lysine, threonine, methionine, and cysteine (3.7 ± 1.4%), RNA processing and modification (3.4 ± 0.7%), and transcription (2.9 ± 0.6%). NMDS analysis was performed based on the abundance of functions classified at the level 2 subsystems (as opposed to level 1 described above). PERMANOVA-tests showed no significant difference in functional profiles between MBL and water column or between daytime and nighttime MBL (PERMANOVA *p* > 0.05, Figure 3B).

### Environmental predictors of dominant taxa and functions

**Figure 4:**
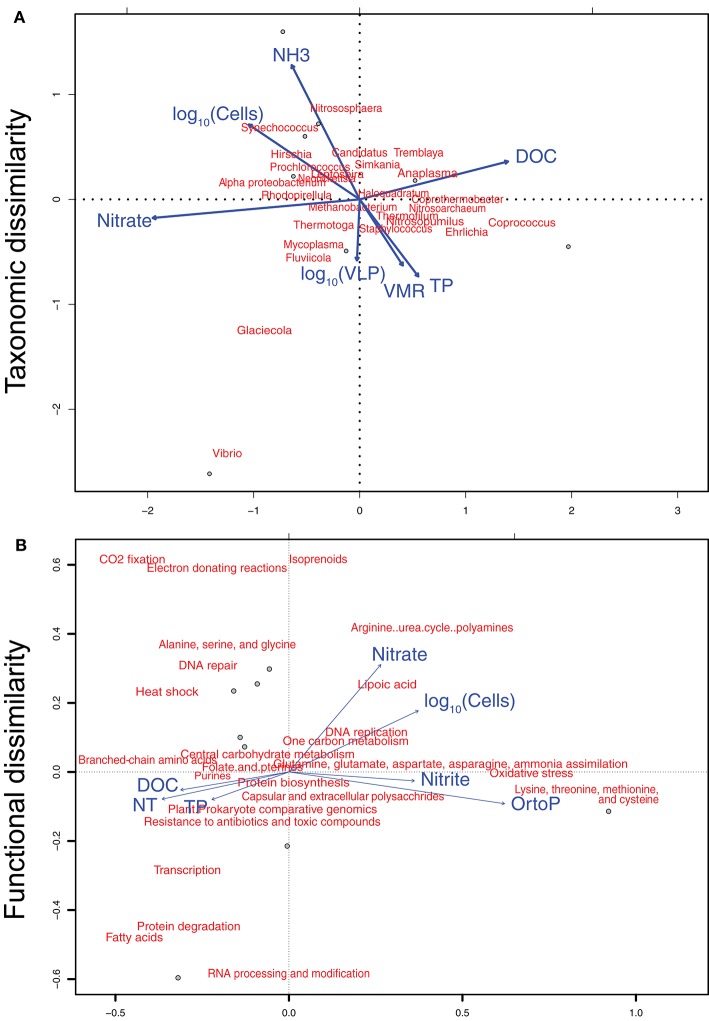
**Canonical correspondence analysis of environmental variables and (A)** dominant genera, and **(B)** dominant functions. Environmental variables used in the analysis are shown in Figure 1 and Table 2. Dominant genera and functions were defining as those with at least 1% abundance. Twenty-five genera and twenty-six functions were utilized in the analysis, summing up to a total of 70.6 and 51.9% of taxonomically and functionally annotated reads, respectively.

We investigated if taxonomic profiles in both MBL and water column samples were determined by nutrient concentrations. We performed these analyses with 26 dominant genera, defined as those with at least 1% relative abundance, which contributed to a total of 70.6% of the community composition. The relationship between environmental variables and dominant taxa can be visualized in the Canonical Correspondence Analysis (CCA) plot (Figure 4A). DistLM analysis tested the contribution of each environmental variable to the dissimilarity among dominant community members. DistLM showed that nitrate was the best predictor of taxonomic dissimilarity (40% of community dissimilarity, *p* = 0.019). Following, DOC contributed to 27%, prokaryotic abundance and source (MBL or water column) each contributed with 17.8%, time of collection with 14%, and total phosphorus with 13.9%. All the other variables contributed to 10% or less the community dissimilarity. We tested significant correlations between nitrate, which had the highest explanatory proportion, and each taxon. *Coprothermobacter* was negatively correlated with nitrate (*r* = −0.91, Holm's post-test correction *p* = 0.04). We then performed a reverse DistLM-test where taxonomic dissimilarity was tested as predictor for environmental variable dissimilarity. *Synechococcus* was the genus explaining the highest proportion of environmental variable dissimilarity (25%, *p* = 0.04).

Analyses of relationships between environmental variables and dominant functional profiles were performed on 25 SEED level 2 subsystems, each one with abundances of at least 1%, contributing to a total of 51.9% of all functionally annotated reads. Correlations between dominant functions and environmental variables can be visualized in the CCA (Figure 4B). DistLM analysis showed that dissimilarity among functional profiles was poorly explained by environmental variables. Orthophosphate, nitrate, and cell abundance were the variables contributing to the highest proportion of explanation (45, 29, and 24%, respectively), however *p*-values were not significant (0.08, 0.1, and 0.1, respectively). Accordingly, the reverse test where functional profiles were tested as predictors of environmental variables showed no significant functions.

## Discussion

Current coral microbiome studies are mostly focused on finding species-specific associations across geographical patterns and disturbance response (Hester et al., 2015; Bourne et al., 2016; Hernandez-agreda et al., 2016). Most studies investigating temporal variability in coral microbiomes are focused on relatively long time scales, weeks to months, with little information available on temporal variability at the diel scale (Glasl et al., 2016; Zaneveld et al., 2016). This contrasts with the strong diurnal variation imposed by coral physiology (Sorek et al., 2014). Here, we aimed to fill this gap by analyzing microbial dynamics on the MBL above the coral *M. braziliensis* at the diel scale. We found no evidence for a diel pattern in bacterial taxonomic and functional profiles at the time scale sampled, and observed that the variability of taxonomic profiles correlated with nutrient concentrations, suggesting that the coral MBL was more influenced by water column dynamics than by coral physiology. This result is consistent with previous findings in water column bacterial communities in the Great Barrier Reef, and supports the hypothesis that diurnal patterns in community composition are related to planktonic processes rather than to benthic–pelagic coupling (Sweet et al., 2010).

The dominant taxa observed in *M. braziliensis* MBL belong to *Proteobacteria, Firmicutes, Thaumarchaeota*, and *Cyanobacteria* phyla, which were previously reported as abundant in *M. braziliensis* mucus, along with *Planctomycetes* and *Bacteroidetes* (Reis et al., 2009; Garcia et al., 2013). Other coral species within the *Mussismilia* genus, however, were previously shown to harbor distinct bacterial community profile even at the phylum level, with higher abundance of *Actinobacteria, Acidobacteria, Lentisphaera*, and *Nitrospira* (Castro et al., 2010). This result shows that both the MBL sample and the water above are under influence of coral mucus and support the hypothesis of stable interactions between microbes and the *Mussismilia* genus (Carlos et al., 2013; Fernando et al., 2014). Growing evidence suggest that different coral species select for specific bacterial assemblages, even at large geographic scales (Hernandez-agreda et al., 2016; Neave et al., 2016). Stable members of the bacterial community were found in low abundance in Porites corals across the Pacific, while abundant members were often sporadic (Ainsworth et al., 2015; Hester et al., 2015). Likewise, rare or low-abundance prokaryotic families drive the differences in microbial community composition between depths within the depth generalist coral *Stephanocoenia intersepta* (Glasl et al., 2017). Microbial community associated with the coral *Seriatopora hystrix* correlated more with habitat than with coral host genotype (Pantos et al., 2015). The lack of diurnal differences observed here may be a result of abundant, sporadic members that do not respond to quick changes in coral physiology compared to stable, low abundance members. We predict that stable members of the holobiont that co-evolve with their hosts would respond to changes in host physiology by synchronizing growth and metabolism. This type of relationship is not expected in transient members that are highly subject to stochastic processes.

Microbial association with corals can be shaped by host genotype and environmental features (Rohwer et al., 2002; Zilber-Rosenberg and Rosenberg, 2008). Although our sampling procedure targets microbes from the MBL, predicted to have a relatively weak relationship with the coral compared to tissue-associated microbes, we observed high abundance of typical symbiotic genera such as *Anaplasma, Hirchia, Neoricketsia, Mycoplasma*, and *Ehrlichia*. These groups comprise obligate endosymbionts of some arthropods and have reduced genomes that retain only essential functions, often including genes that serve the hosts (López-Madrigal et al., 2011; McCutcheon and Moran, 2011). It is possible that the sequences observed here came from obligate coral symbiont species belonging to these genera, but not yet described. These microbes could be both naturally expelled from the coral surface during mucus exudation or we could have accidentally sampled fragments of coral tissue during the MBL pumping. Although bacterial assemblages differ between compartments within the coral holobiont, endosymbiotic microorganisms are found in coral mucus samples collected by the milking method, showing that differentiating between these compartments requires fine-scale sampling procedures (Sweet et al., 2011a).

Association between specific microbial community functional profiles and environmental conditions are consistently observed over time and space, regardless of species composition (Huttenhower et al., 2012). The highly similar functional profiles found here in both diurnal and nocturnal MBL, and lack of correlation with short-term fluctuation in nutrient concentration corroborates the idea of a functional profile characteristic of the studied reefs. Functions related to carbohydrate metabolism, amino acids, and protein metabolism were the most abundant, as observed before in water column microbial metagenomes of the Abrolhos region (Bruce et al., 2012). This profile differs from those of pristine coral reefs in the Pacific, where respiration and virulence were the most abundant functions (Dinsdale et al., 2008a,b). These differences may, however, be a result of method variability, from DNA extraction to sequencing (Wood-Charlson et al., 2015).

Despite the lack of a diel pattern in nutrients and microbial abundance during the sampled period, we identified a synchronized change in total prokaryotic and viral abundance that indicated a lytic event. Studies of short time scale variation in viral abundances in marine environments have conflicting results, and most of them did not display diel cycles (Jiang and Paul, 1994; Winter et al., 2004; Winget and Wommack, 2009). Likewise, little empirical evidence exists for Kill-the-Winner dynamics in complex mixed communities (Thingstad, 2000; Knowles et al., 2016). Interestingly, pico- and nanoeukaryotes abundance were negatively correlated with total viral abundance (*r* = −0.61 and −0.65, respectively). This suggests that a significant fraction of the active community might be comprised of eukaryotic viruses, despite the fact that these viruses comprise only about 10% of total the viral community (Brussaard et al., 2010; Silveira et al., 2015). It was not possible to differentiate groups V1, V2, and V3 in our samples, which correspond to bacteriophages or eukaryotic viruses, preventing to conclusively determine if the largest variance was indeed in the eukaryotic virus group (Brussaard, 2004; Brussaard et al., 2010).

The lack of diel patterns and the correlations between taxonomic dissimilarities and nutrients suggest that water flow over corals determines microbial community composition in the MBL, rather than diel switches in coral physiology. Community assembly patterns observed here in the MBL, and before in the coral holobiont, indicate that stochastic processes such as migration or nutrient fluctuations due to hydrodynamics are important forces shaping the short-term fluctuations in the microbiome, as opposed to deterministic processes (Sloan et al., 2006; Hester et al., 2015). This pattern has been observed in several microbial communities and can be explained by a stochastic neutral community model (Sloan et al., 2006). It is also possible that significant diurnal changes in the community happen in the coral holobiont and in the diffusive boundary layer at the millimeter scale. The diffusive boundary layer of *S. pistillata* is significantly thinner when a 5 cm/s current is applied (Shashar et al., 1993). To address whether diurnal patterns in community composition occur on coral diffuse boundary layer, tank, and field approaches that target spatially-defined sampling at the scale of millimeters, reduce water flow, or amplify the effect of coral physiology are necessary.

In conclusion, we show that the microbial community in the MBL of the coral *M. braziliensis* do not display significant diurnal patterns. Instead, this community is similar to the water column 1 m above the corals and responds to environmental fluctuations in nutrient concentrations. Our results suggest that despite coral mucus contribution to the MBL community composition, the overlying water currents contribute more to short term variability in community assembly than diurnal changes in coral physiology. These results expand the view of short-term temporal dynamics of coral microbiome, often bypassed in studies on coral health and reef ecology.

## Author contributions

CS, GG, and FT designed the study; CS, GG, FC, GS, JH, LO, AC, CR, CT, RF, RE, and ED generated and analyzed the data; CS and FT wrote the manuscript and all authors contributed to revisions.

### Conflict of interest statement

The authors declare that the research was conducted in the absence of any commercial or financial relationships that could be construed as a potential conflict of interest.
